# Human and Algorithmic Predictions in Geopolitical Forecasting: Quantifying Uncertainty in Hard-to-Quantify Domains

**DOI:** 10.1177/17456916231185339

**Published:** 2023-08-29

**Authors:** Barbara A. Mellers, John P. McCoy, Louise Lu, Philip E. Tetlock

**Affiliations:** 1 Department of Marketing, University of Pennsylvania; 2 Department of Marketing, Stanford Business School, Stanford University; 3 Management Department of Wharton Business School, University of Pennsylvania

**Keywords:** predictions, forecasts, artificial intelligence, algorithms, clinical versus statistical prediction debate

## Abstract

Research on clinical versus statistical prediction has demonstrated that algorithms make more accurate predictions than humans in many domains. Geopolitical forecasting is an algorithm-unfriendly domain, with hard-to-quantify data and elusive reference classes that make predictive model-building difficult. Furthermore, the stakes can be high, with missed forecasts leading to mass-casualty consequences. For these reasons, geopolitical forecasting is typically done by humans, even though algorithms play important roles. They are essential as aggregators of crowd wisdom, as frameworks to partition human forecasting variance, and as inputs to hybrid forecasting models. Algorithms are extremely important in this domain. We doubt that humans will relinquish control to algorithms anytime soon—nor do we think they should. However, the accuracy of forecasts will greatly improve if humans are aided by algorithms.

In 1954, Paul Meehl wrote an influential book that grappled with the question: Will we make more accurate forecasts—in selecting job applicants, determining medical diagnoses, or making parole decisions, for example—if we base our predictions on clinical and intuitive judgments or on statistical models? Meehl (1954) stipulated the ground rules for a fair competition. Both methods should have access to the same data—and algorithms should be cross-validated in an independent sample to avoid overfitting. Meehl gathered as many studies as he could that satisfied these criteria and discovered that algorithms almost always tied or beat human competitors.

## The Clinical Versus Statistical Prediction Debate

Over the last several decades, statistical models have extended their leads in domains such academic success (Dawes, 1971; Schofield & Garrard, 1975; Wiggins & Kolen, 1971), cancer-survival times (Einhorn, 1972), myocardial infarctions (Goldman et al., 1988; Jakubowski, 1988; K. L. Lee et al., 1986), and neuropsychological disorders (Leli & Filskov, 1984; Wedding, 1983). Meta-analyses reached similar conclusions. Grove et al. (2000) surveyed the literature in psychology, medicine, forensics, and finance and found that statistical methods had a small but consistent advantage over human intuitions (*d* = 0.12). Next, Ægisdóttir et al. (2006) compared the intuitions of mental-health practitioners to statistical models and found that statistical models reliably edged out human intuitions. Several scholars have proposed a simple division of labor between humans and models; humans should identify the predictor variables, and models should be used to aggregate information (Khosrowabadi et al., 2022; Lu et al., 2021; Önkal et al., 2009).

Meehl built his case by pitting the predictive power of human forecasters against statistical models with access to the same cues as those available to humans. Using correlations that were often constructed from individual predictions and outcomes, Meehl argued that humans were usually less accurate than models. The notion of aggregating individual predictions to obtain more reliable and valid forecasts—the wisdom-of-the-crowd hypothesis—was not the focus of the debate.

Over the last few decades, evidence has accumulated that crowd predictions are often wiser than most individual predictions (Sunstein, 2006; Surowiecki, 2004). This well-established finding has sparked follow-up questions. How big does the “crowd” need to be? Studies on crowd size suggest that the answer is surprisingly few, as long as the individuals are carefully selected. For example, Mannes et al. (2014) introduced a select-crowd strategy that ranks individuals on the basis of their ability to answer a set of relevant questions. The average of the top five individuals (what they call a “select” crowd) provided accurate predictions over a wide range of domains.

Can one find the most accurate individuals even before questions are resolved? Himmelstein, Budescu, and Ho (2023) and Atanasov, Karger, and Tetlock (2023) found that individuals who made judgments closest to the aggregate forecast were among the top performers, a prediction made by *cultural consensus theory* (Batchelder & Anders, 2012). Himmelstein et al. and Atanasov et al. demonstrated that intersubjectivity scores are frequently good estimates of predictive skill, and they can be obtained before events have been resolved. Furthermore, the selection of a small set of forecasters based on intersubjective accuracy could yield forecasts as accurate as those based on large crowds.

What is the best algorithm for combining individual judgments? Simple algorithms, such as means, medians, and weighted averages, have proved impressively robust and hard—but not impossible—to beat (Clemen & Winkler, 2007; Fisher, 1981; Winkler et al., 2019). For instance, Budescu and Chen (2015) developed an ingenious way of aggregating forecasts called the *contribution-weighted model*. Individuals receive weights that reflect the degree to which they contributed to the accuracy of each question’s aggregate forecast. The contribution of any individual is obtained by recalculating the aggregate forecast without that person for each person on every question. Weights change dynamically as time passes and events are resolved. Only persons with positive contributions are in the final aggregate. E. Chen et al. (2016) showed that this aggregation rule is remarkably effective.

## Geopolitical Forecasting

Is the overall process of making predictions generally similar across domains? Do psychiatrists who predict the well-being of institutionalized patients do essentially the same thing as intelligence analysts who predict political crises? In both cases, humans play an indispensable role in distilling diverse, hard-to-quantify cues from the environment and translating their hunches into probabilities. However, psychiatrists have access to objective measures from independent samples of thousands of patients, from blood-chemistry panels and neuro imaging to intelligence tests, work history, and staff ratings. Psychiatric data can be funneled into multiple regressions and put in direct competition with human forecasters in out-of-sample tests. As mentioned earlier, humans tend to lose these competitions, which raises a division-of-labor question: Why not let humans identify key predictor variables and let machines do the combinatorics and prediction-error minimization?

Intelligence analysts who make geopolitical predictions face a very different situation. They have access to some data, but rarely the data they want. Suppose they want to predict the spillover effects from the Syrian civil war. Intelligence analysts do not get to observe thousands of versions of the Syrian civil war. History only unfolds once—so powerful regression models are difficult to build. The most analysts can do is match the Syrian civil war against previous civil wars, and such conflicts are less frequent and more heterogeneous than psychiatric patients within disease categories.

In short, reference classes of relevant historical precedents are elusive. Consider the question of whether Russia will use nuclear weapons in the Ukraine. Russian threats of nuclear war have precedents in the Cuban Missile Crisis, but circumstances in 2023 differ from those in 1962. And even classifying actual outcomes can be difficult for analysts; debate still continues in 2023 about whether the uneasy truces in regions of Syria qualify as a cessation of hostilities.

Evidence for policy decisions often comes from counterfactual conjecture, not factual data (Tetlock & Belkin, 1996). In the geopolitical domain, policymakers imagine what outcomes might have occurred under different circumstances and draw on their favorite causal theories to fill in the blanks. Furthermore, many critical events, such as nuclear war, bioterrorism, and pandemics, have low probabilities of occurrence. Models are hard to build because there are few observations that are difficult to classify.

### Tournaments to improve human forecasts

Does that mean that forecasting in the geopolitical domain does not benefit from algorithms? The answer is no. We discovered the power of algorithms in geopolitical forecasting between 2011 and 2015 when the U.S. intelligence community commissioned a series of forecasting competitions. The Intelligence Advanced Research Projects Activity (IARPA), the research wing of the intelligence community, funded five university groups to develop approaches for predicting hundreds of outcomes from scenarios that intelligence analysts routinely confront: the risk of naval clashes in the South China Sea, or whether Spanish–German bond yield spreads would rise, or what spillover effects would occur from the Syrian civil war.

The ground rules for the tournaments were simple: Researchers could deploy whatever mix of strategies they deemed best for winning. The winner was the group that maximized accuracy as defined by the Brier scoring rule (Brier, 1950). Brier scores are sums of squared deviations between probability predictions and actual events (coded as 1 if the event occurred and 0 otherwise).

Some of the authors of this paper were part of a research group called the Good Judgment Project. We did not have access to statistical models or forecasts for most of the tournament questions. Instead, we obtained forecasts from volunteers all over the world. We discovered that several strategies improved human forecasts, and these strategies allowed us to win the tournament each year for 4 years (Chang et al., 2016; Mellers et al., 2014; Mellers, Stone, Atanasov, et al., 2015; Satopää, Baron, et al., 2014; Satopää, Jensen, et al., 2014; Satopää et al., 2017). Here, we briefly discuss these strategies for identifying talented forecasters, training them in probabilistic reasoning, eliciting predictions, organizing crowds, and aggregating forecasts.

#### Identifying talent

At the start of each tournament year, we gave volunteers a battery of psychological and political knowledge tests. Using these data, we could investigate the correlates of forecasting accuracy. We hypothesized and found evidence that individuals with greater skill at inductive reasoning, cognitive control, and numerical reasoning were more accurate forecasters. Cognitive style was also predictive: More actively open-minded forecasters were more accurate (Haran et al., 2013). And, not surprisingly, more politically knowledgeable forecasters were more accurate.

#### Training

Each year, we developed interactive online training modules to help forecasters improve their judgments. These training modules instructed people to consider multiple reference classes; average predictions from models, polls, or expert panels if they had more than one; extrapolate over time when variables were continuous; and avoid judgmental traps such as overconfidence, base-rate neglect, and the confirmation bias. Training improved forecaster accuracy by 6% to 11% each year relative to untrained forecasters (Chang et al., 2016).

#### Elicitation

When we confronted the question of how best to elicit wisdom from the crowd, we found competing ideas in the literature. Therefore, we experimentally tested different methods by randomly assigning forecasters to different conditions. One elicitation method was continuous prediction polling—asking forecasters to predict events with probability judgments and update their beliefs as often as they wished over an extended period of time, with incentives for providing the most accurate forecasts and quick feedback after events resolved.

Another method is a prediction market, often preferred by economists. In prediction markets, forecasters act as traders who place bets on future events (Wolfers & Zitzewitz, 2004). A contract can pay $1 if the event happens, and $0 otherwise. If the current price is $0.60, the supply-demand equilibrium implies that the event has a 60% chance of occurring. We explored several types of prediction polls and several types of prediction markets (Atanasov et al., 2017; Dana et al., 2019). In the process, we learned that the best prediction polls combined with the best aggregation algorithms often produced forecasts that were just as accurate or even more than prediction markets (Atanasov et al., 2022; Y. Chen & Pennock, 2010; Elliott & Timmermann, 2013; Mellers & Tetlock, 2019).

#### Crowd interaction

If forecasters in a crowd make probability predictions about events over time, should that crowd interact to generate the most accurate predictions? Or should individuals work alone? To find out, we randomly assigned forecasters to conditions in which they either made independent forecasts or interacted online sequentially in teams of roughly 10 to 15 people. Forecasters who worked together were more accurate than independent forecasters each year for 4 years. The opportunity for discussion allowed forecasters to motivate one another, share news, exchange rationales, and debate the likelihood of events.

### Aggregation algorithms

The strategies described above improved the accuracy of human predictions, but not by means of algorithms. Next we turn to the first way in which algorithms played a crucial role in boosting the accuracy of geopolitical forecasts—aggregating the wisdom of the crowd. Aggregation algorithms gave us a critical advantage in the tournaments (Mellers, Stone, Atanasov, et al., 2015; Mellers, Stone, Murray, et al., 2015; Tetlock & Gardner, 2015).

We began with simple algorithms—means and medians—that were selected on the basis of their ability to reduce noise via error cancellation when forecasters worked alone. But as the years progressed, our algorithms grew in complexity and extremity. We adjusted means to give greater weight to more recent forecasts, building on the idea that forecasters who updated their predictions were more likely to have better information, thus improving the signal-to-noise ratio. Furthermore, we assigned greater weight to those with better track records.

Finally, we added an extremizing transformation (Baron et al., 2014; Satopää, Baron, et al., 2014; Satopää, Jensen, et al., 2014; Ungar et al., 2012). The idea of extremizing aggregate probabilities is not new (e,g., Erev et al., 1994). It means transforming the aggregate away from .5. A weighted mean forecast of .7 might be pushed upward to .9, and a weighted mean forecast of .3 might be pushed downward to .1. This raises the question: How does one know when and how much to extremize a forecast?

Satopää et al. (2017) developed a partial information framework to answer that question. Forecasters collect information from a variety of sources, including the Internet, friends, and experts. Suppose we have two crowds of forecasters. In the first crowd, forecasters look at identical pieces of information. This means complete overlap of signals and noise among forecasters, as if there is only one forecaster. In this case, there is no need to extremize. In the second crowd, forecasters look at entirely different sources of information that are both reliable and valid. Simply averaging their forecasts would not reflect the greater amount of information underlying the aggregate. Here, extremizing would greatly improve the accuracy of the aggregate forecast.

**Fig. 1.**
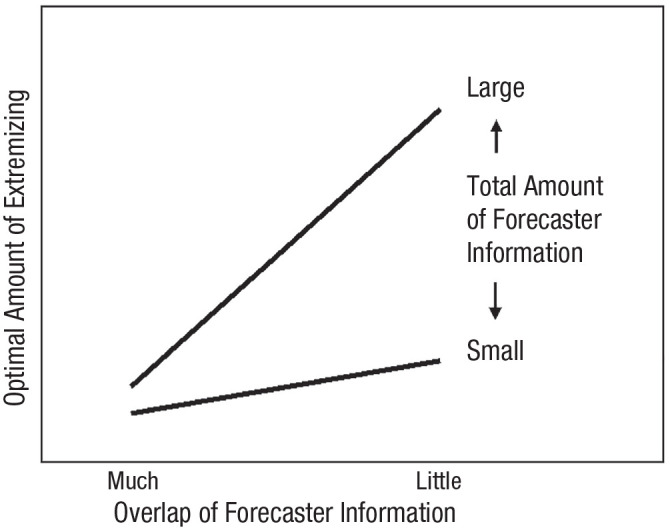
Overlap of Forecaster Information

A stylized version of the optimal degree of extremizing is shown in Figure 1. Extremizing depends on two factors—the overlap of forecaster information (shown on the *x*-axis as “much” or “little”) and the total amount of forecaster information (shown with lines indicating “large” or “small”). Extremizing is most beneficial when the total amount of information is large and forecasters have little overlapping information.

#### Factors that influence extremizing in the partial information framework

We could test the partial information framework by comparing how much to extremize aggregate forecasts coming from different conditions of the Good Judgment Project. When we aggregated a large number of independent forecasters, accuracy was greatest when the aggregate forecast was heavily extremized. When regular forecasters worked in teams of 15 people, each team had less total information than the large crowd, and interactions among team members increased information overlap. In this case, less extremizing was needed. Finally, there was no reason to extremize teams of superforecasters who were the most accurate 2% of forecasters each tournament year. Although superforecasters gathered a formidable amount of information, they shared and discussed it widely. The overlap of forecaster knowledge made extremizing completely unnecessary.

Powell et al. (2022) took a different approach to extremizing. They also used data from the Good Judgment Project and showed that a skew-adjusted extremized-mean algorithm could successfully boost accuracy. The algorithm made more aggressive predictions when it detected fat-tailed distributions of forecasts that implied contrarian-minority opinions. Powell et al. could increase accuracy despite the fact that they had no information about which forecasters were in the fat tails, whether their track records were weak or strong, whether they possessed relevant subject-matter expertise, and indeed whether they were even aware of where they fell in the distribution of other forecasters.

Aggregation algorithms must adjust to the environment and the forecasters. There may be a steep cost to accuracy if one applies a pure noise-reduction aggregator, such as a mean, to predictions that vary because forecasters draw on distinctive pockets of information. And there may also be a steep cost to accuracy if too much extremizing is applied to forecasts of events that are driven by power laws—and available historical base rates are deceptively calm. To better understand when and where to extremize, we recommend moving beyond horse-racing comparisons of aggregators that minimize errors to investigating aggregation algorithms in different situations in which individuals interact in different ways (Davis-Stober et al., 2014; Soll & Larrick, 2009).

### Understanding forecasting strategies

A second way in which algorithms were important in the geopolitical-forecasting domain was by helping us discover the reasons why our interventions improved accuracy. Satopää et al. (2021) developed a Bayesian framework called the bias-information-noise (BIN) model, which is based on the notion that prediction accuracy improves in three ways: bias reduction, noise decline, and information acquisition. The BIN model assumes that forecasters confront a vast signal universe populated by cues bearing on a given target event. The target event occurs only after the cumulative signal becomes positive. Accuracy depends on the perceptiveness of the forecasters and the thoroughness with which they sample from this universe.

Satopää et al. (2021) used the BIN model to explore how training, teaming, and tracking (or placing the top 2% of forecasters in elite teams) boosted predictive accuracy in the Good Judgment Project. We compared variation in forecasts over time between trained and untrained forecasters, between team forecasters and independent forecasters, and between superforecasters in teams and regular forecasters in teams.

Our working hypotheses were that training would reduce cognitive biases, such as base-rate neglect, overconfidence, and the confirmation bias, by encouraging forecasters to adopt an outside view (Kahneman, 2011); teaming would prevent biases from groupthink or failures to share information; and tracking would allow the most insightful forecasters (relative to regular forecasters) to work together to discover more valid signals, reduce bias, and decrease noise. To our surprise, the BIN model revealed that over 50% of the increase in accuracy derived from all three interventions was due to noise reduction. (See Kahneman et al., 2021, for a discussion of the pervasiveness of noise.)

Satopää et al. (2022) also extended the BIN framework to examine how the aggregation algorithm applied to multiple forecasters improved accuracy relative to a typical individual forecaster. Satopää et al. investigated the aggregation methods with some “unsupervised algorithms,” such as simple averaging, that do not require data on forecasters’ past performance. The BIN model showed that the benefits of averaging multiple forecasts compared with a typical individual forecaster were almost entirely due to noise reduction.

Another unsupervised algorithm that does not need past data on participants is a prediction market. In prediction markets, the market price reflects the crowd belief that an event will occur. Traders with different beliefs exchange contracts so that prices reflect the collective chances of future events. When Satopää et al. (2022) compared the market’s equilibrium price to that of a typical individual trader, the market equilibrium was more accurate by reducing noise, decreasing bias, and enhancing information (Atanasov et al., 2017; Mellers & Tetlock, 2019).

### Hybrid algorithms

Most studies in the clinical- versus statistical-prediction debate did not examine the accuracy of hybrid models that relied on both statistical and human inputs. This oversight was a missed opportunity, because the debate was about relative accuracy, not absolute accuracy. Hybridization has enormous potential for both statistical reasons (greater accuracy than humans alone) and psychological reasons (humans can still be in charge).

Today, hybrid algorithms are used in many domains. IARPA funded another forecasting project that used hybrid methods to predict geopolitical events. One of the research groups developed a hybrid model to predict time-series questions such as, “In November, 2019, what will the price of gasoline be in Kenya in the Nairobi market?” (Benjamin et al., 2023). Forecasters could interact with algorithmic predictions, and the weights of human versus algorithmic predictions depended on the similarity of predictions and the past accuracy of methods. That approach proved to be more accurate than human forecasters alone.

In the epidemiological domain, Atanasov, Joseph, et al. (2023) developed a hybrid model to predict the results of clinical trials for vaccines. Forecasters defined their own base rates given clinical-trial databases and made probability estimates. Inputs were combined statistically using a random-forest regression technique. The model was evaluated in two 6-month forecasting tournaments with questions about the success of clinical trials for vaccines and various treatments for COVID-19 and other infectious diseases. The hybrid model outperformed the statistical model alone and demonstrated that human forecasters added value to a statistical approach, producing relatively good forecasts in quite challenging settings.

In the political realm, Graefe et al. (2014) examined predictions of six U.S. presidential elections from 1992 to 2012. They collected inputs from experts, polls, models, and the Iowa Electronic Markets. By averaging similar inputs (i.e., multiple polls) and using these inputs to predict election results, Graefe et al. discovered that the hybrid method was more accurate than every type of input alone.

In psychological research on face recognition, Phillips et al. (2018) tested the accuracy of experts and super-recognizers against machine-learning algorithms at identifying human faces. By combining the most accurate models with the most accurate facial examiners, predictions were more accurate than combinations of humans or combinations of algorithms.

In medical diagnoses, Patel et al. (2019) developed a collective intelligence platform called Swarm that combined the predictions of networked radiologists working together in real time to diagnose pneumonia from chest radiographs. The accuracy of interacting radiologists was compared with that of radiologists working alone and with two deep-learning artificial-intelligence (AI) models. The greatest accuracy was achieved by combining the predictions of radiologists working together with the predictions of the AI systems. And in another medical study, Tschandl et al. (2020) examined the accuracy of human predictions and image-based AI predictions to diagnosis skin cancer. When image-based AI systems were used in conjunction with physicians’ diagnoses, accuracy was greater than either AI predictions or physicians’ diagnoses alone.

In the business realm, Blattberg and Hoch (1990) used a hybrid model to predict catalog sales and coupon-redemption rates. The average of statistical predictions and managers’ intuitive predictions was more accurate than the predictions of either method alone. Blattberg and Hoch surmised that the models were too consistent and the managers were too flexible, making hybrids an ideal mix. We believe hybrid models such as these are the likely next steps to a world where algorithms are trusted, acceptable, and widespread.

## Future Directions

The list of unanswered questions about the roles of humans and algorithms is long—but we see four promising avenues for future research.

### What variables predict high-performing individuals?

The answer to this question includes a wide array of cognitive abilities and cognitive styles as well as measures of subject-matter knowledge, effort, and working patterns (Mellers, Stone, Atanasov, et al., 2015). Some of the best predictors are frequent incremental-belief updating (Atanasov et al., 2020), granular use of the probability scale (Friedman et al., 2017), measures of logical coherence (Mellers et al., 2017) and scores that capture intersubjective accuracy, awareness of the viewpoint space, and skill at predicting the judgments of other forecasters (M. Lee et al., 2018; Radas & Prelec, 2019).

We recommend extending the search for skilled forecasters to dispositional variables (i.e., cognitive abilities and cognitive styles), situational variables (i.e., teams, training, expertise) and motivational variables (i.e., engagement, efficiency), with the goal of building up a diversified portfolio of predictors of forecasting skill that apply to a variety of different domains.

As algorithms become more widely used in complex domains, other types of individual skills—beyond the ability to produce accurate forecasts—may be worth considering. Individuals who can pose diagnostic forecasting questions, identify novel reference classes, or understand how a forecasting model is likely to err may be especially valuable. Research on the measurement and prediction of these skills is another useful avenue of future research.

### What variables predict high-performing teams?

Teams do not always work well together. Sometimes members avoid effort and rely on members who are more conscientious (Latané et al., 1979). Another problem is that teams can suffer from groupthink—the tendency to make decisions on the basis of consensus with little regard for critical evaluation of evidence or alternative options (Esser, 1998; Janis, 1982). Individuals in groups can form *information cascades*, in which each person makes the same decision as the last in a sequential fashion (Bikhchandani et al., 1992). Groups may also disregard unique information, unshared information, or hypothesis-disconfirming information (Kerr et al., 1996; Stasser & Titus, 1985; Sunstein, 2006). When does collaboration help predictive accuracy and when does it hurt? Is there some way to know which teams will benefit from collaboration?

Suppose the task is to predict an event or to estimate an unknown quantity, such as future stock prices, geographic distances, or historical dates. Silver et al. (2021) discovered a variable that predicts when collaborative interaction will help accuracy. They called it *collective confidence calibration*, an index that robustly predicts when discussion is beneficial relative to initial independent estimates. Collective calibration occurs when—prior to discussion—more accurate team members are more confident and less accurate team members are less confident. Why? Team members listen to the most confident individuals, especially when team members are strangers. When confidence and knowledge are positively associated within a team, more knowledgeable members are likelier to be influential. Teams should strive to be collectively calibrated. Interventions with individual or team-level training are another important line of future work.

### How should the crowd be configured?

Many researchers have stressed the need for diversity in crowds (Davis-Stober et al., 2014; Page, 2007). The argument for diversity over ability is that a single perspective rarely has a monopoly on truth. Insights are often distributed. By contrast, the superforecasting research program was explicitly elitist: accuracy was greatest when the top performers worked together in elite teams. Is it possible to reconcile these perspectives?

Imagine two teams, each with two observers who make predictions about the Russia–Ukraine war. In one team, both forecasters have strong track records and subscribe to a deterrence theory of international conflict that posits the surest way to achieve peace is by possessing the military capacity and projecting the political will to resist aggressors. In the other team, both forecasters have less impressive track records, and each subscribe to different theories of conflict. One is a *deterrence theorist*, and the other is a *conflict-spiral theorist* who believes that the surest way to achieve or restore peace is reduce misperceptions in which each side exaggerates the other’s hostile intent. Designers of elitist algorithms will bet on the stronger track-record forecasters with similar views, whereas designers of more egalitarian algorithms will bet on the forecasters with clashing views, even though the forecasters have weaker track records.

The BIN framework can inform this debate by gauging the degree to which the predictive-accuracy boost in the team that relies on two strong-track-record forecasters exceeds the accuracy boost from the team with two regular forecasters with clashing schools of thought. Is the risk of elevated bias from relying on one school of thought greater than the risk of elevated noise from drawing on discordant schools of thought? Does less noise from reliance on one school of thought outweigh less bias from drawing on clashing schools of thought (which may create more potential for correcting each other’s misconceptions)?

Using the BIN framework and supervised algorithms (with access to the track records of forecasters who vary in skill and schools of thought), we can clarify when elitist or egalitarian prescriptions are likelier to come closer to the truth and why algorithms work well in different environments. This is another avenue in which research is needed.

Another way to reconcile debates about crowd configurations is to have forecasters make predictions of others’ predictions. For example, consider the Bayesian truth serum (Prelec, 2004; Weaver & Prelec, 2013), which incentivizes honesty even when events are unresolvable, and the surprisingly popular algorithm (Prelec et al., 2017), which aggregates information across individuals. These methods ask individuals to provide their own answer to a question and also predict the distribution of answers in the crowd. This method is especially helpful when mistakes are widely shared and truly insightful information is known only to a few.

To illustrate the surprisingly popular approach, imagine that respondents are asked, “What is the capital of California?” Some respondents know the answer is Sacramento, but many will say the capital is a larger city, such as Los Angeles. Respondents who say “Sacramento” may be more likely to correctly predict that other respondents will incorrectly select Los Angeles. But respondents who say “Los Angeles” will probably not realize that a small minority will say “Sacramento.” Thus, Sacramento is a response that occurs more often than expected, a clue that it might well be the correct answer.

Diversity of thought can be valuable not only within crowds, but also within individuals. Another hypothesis deserving of more research is that the best forecasters of objective outcomes achieve their status because they have internalized a wider range of perspectives in their private deliberations—in effect, a diverse “crowd within” (Herzog & Hertwig, 2009; Vul & Pasher, 2008). When forecasters take different perspectives, they bring more and better arguments to bear on a judgment. Galesic et al. (2021) demonstrated this idea with predictions of behavior (i.e., voting or vaccination decisions): Forecasters were more accurate when they imagined the views of friends, family, and coworkers. Galesic et al. showed that the 2018 and 2020 U.S. elections were better predicted by social-circle expectations than by traditional polling questions that asked only about the individual’s intentions. See Rothschild and Wolfers (2011) for another example.

### How should humans and algorithms work together?

When it comes to making predictions, humans and algorithms have strengths and weaknesses. Humans may know what questions to ask, what variables may be useful, and what reference classes to use (e.g., the chance of a rainy day in Seattle). Humans can also be flexible about rapidly changing conditions; they may be aware of highly unusual patterns of diagnostic cues that are hard to incorporate into models. Yet humans have distinctive weaknesses. They can be insensitive to relevant factors (e.g., base rates) and sensitive to irrelevant ones (e.g., pseudodiagnosticity; Doherty et al., 1979). Algorithms are less noisy and immune to social or organizational pressures. They can optimally weigh and aggregate evidence in a reliable fashion, but their consistency may result in rigidity.

Research is needed to determine how algorithms can best help humans to reduce or alleviate their weaknesses. We have discussed how algorithms in geopolitical forecasting can aggregate human judgments, partition variance in forecasts, and improve accuracy in hybrid models. These are not the only possibilities, however. Algorithms can also be used to alert forecasters to overlooked sources of useful information, to help them visualize arguments and ideas, to correct reasoning errors in rationales, and to flag changes in quantitative variables that may merit updating forecasts. That is, algorithms can help humans become smarter.

Geopolitical forecasting is profoundly important, but forecasters rarely have access to large data sets of observations and easily quantifiable predictor variables. Therefore, it is hard to apply conclusions from the classic literature on clinical versus statistical predictions to this domain. Replacing human judgment with algorithms is far from straightforward. Future research should examine different forms of hybrid methods, with special emphasis on more rapid identification of forecasting talent, more effective protocols for team deliberation, and increasingly powerful crowd-wisdom aggregators.

## Transparency

*Action Editor:* Mirta Galesic

*Editor:* Interim Editorial Panel
